# Tumor Mutational Burden From Tumor-Only Sequencing Compared With Germline Subtraction From Paired Tumor and Normal Specimens

**DOI:** 10.1001/jamanetworkopen.2020.0202

**Published:** 2020-02-28

**Authors:** Kaushal Parikh, Robert Huether, Kevin White, Derick Hoskinson, Nike Beaubier, Haidong Dong, Alex A. Adjei, Aaron S. Mansfield

**Affiliations:** 1 Division of Medical Oncology, Mayo Clinic, Rochester, Minnesota; 2 Division of Medical Oncology, John Theurer Cancer Center, Hackensack, New Jersey; 3 Tempus Labs, Chicago, Illinois; 4 Department of Urology, Department of Immunology, Mayo Clinic, Rochester, Minnesota

## Abstract

**Importance:**

Tumor mutation burden (TMB) is an emerging factor associated with survival with immunotherapy. When tumor-normal pairs are available, TMB is determined by calculating the difference between somatic and germline sequences. In the case of commonly used tumor-only sequencing, additional steps are needed to estimate the somatic alterations. Computational tools have been developed to determine germline contribution based on sample copy state, purity estimates, and occurrence of the variant in population databases; however, there is potential for sampling bias in population data sets.

**Objective:**

To investigate whether tumor-only filtering approaches overestimate TMB.

**Design, Setting, and Participants:**

This was a retrospective cohort study of 50 tumor samples from 10 different tumor types. A 595-gene panel test was used to assess TMB by adding all missense, indels, and frameshift variants with an allelic fraction of at least 5% and coverage of at least 100× within each tumor. Tumor-only TMB was evaluated against the criterion standard of matched germline-subtracted TMB at 3 levels. Level 1 removed all the tumor-only variants with allelic fraction of at least 1% in the Exome Aggregation Consortium database (with the Cancer Genome Atlas cohort removed). Level 2 removed all variants observed in population databases, simulating a naive approach of removing germline variation. Level 3 used an internal tumor-only pipeline for calculating TMB. These specimens were processed with a commercially available panel, and results were analyzed at the Mayo Clinic. Data were analyzed between December 1, 2018, and May 28, 2019.

**Main Outcomes and Measures:**

Tumor mutation burden per megabase (Mb) as determined by 3 levels of filtering and germline subtraction.

**Results:**

There were significantly higher estimates of TMB with level 1 (median [range] mutations per Mb, 28.8 [17.5-67.1]), level 2 (median [range] mutations per Mb, 20.8 [10.4-30.8]), and level 3 (median [range] mutations per Mb, 3.8 [0.8-12.1]) tumor-only filtering approaches than those determined by germline subtraction (median [range] mutations per Mb, 1.7 [0.4-9.2]). There were no strong associations between TMB estimates and tumor-germline TMB for level 1 filtering (*r* = 0.008; 95% CI, −0.004 to 0.020), level 2 filtering (*r* = 0.018; 95% CI, 0.003 to 0.033), or level 3 filtering (*r* = 0.54; 95% CI, 0.36 to 0.68).

**Conclusions and Relevance:**

The findings of this study indicate that tumor-only approaches that filter variants in population databases can overestimate TMB compared with germline subtraction methods. Despite improved association with more stringent filtering approaches, these falsely elevated estimates may result in the inappropriate categorization of tumor specimens and negatively affect clinical trial results and patient outcomes.

## Key Points

**Question:**

Are filtering approaches an appropriate alternative to germline mutation subtraction for calculating tumor mutational burden (TMB)?

**Findings:**

In this cohort study of 50 tumor samples comparing TMB calculated using 3 filtering approaches with germline-subtracted TMB, no strong association was found between TMB calculated using any filtering method and germline-subtracted TMB.

**Meaning:**

These findings suggest that tumor-only methods of calculation may falsely overestimate TMB, potentially affecting patient care and treatment outcomes adversely; germline subtraction may more accurately measure TMB.

## Teaser

This cohort study investigates whether tumor-only filtering approaches overestimate tumor mutation burden.

## Introduction

Immune checkpoint inhibitors (ICI) are approved for the treatment of many malignant neoplasms, but there is considerable debate as to how accurately biomarkers predict benefit from these agents. While programmed cell death ligand 1 expression has been used to predict ICI response, its heterogeneity and dynamic expression patterns complicate its use.^1^ Tumor mutation burden (TMB), defined as the number of nonsynonymous mutations per megabase (Mb) of tumor tissue DNA, is an emerging factor associated with survival for patients treated with ICI.^2^ Data suggest that TMB is a surrogate for the quantity of tumor neoantigens recognizable by the adaptive immune system, which can target and eliminate tumor cells on neoantigen detection.^3^ Although, to our knowledge, there is no standardized methodology to calculate TMB, the subtraction of germline variants from paired tumor-normal sequencing data provides the most accurate determination of somatic mutations and therefore the most accurate TMB calculation. Because the identification of germline variants requires sequencing of a normal (ie, germline) sample (saliva or blood), which increases the cost of testing, TMB is often calculated from tumor-only sequencing approaches that filter common germline variants reported in population databases. Here, we present discrepancies in TMB measurements by comparing the paired tumor-normal to germline-subtracted somatic mutational load with 3 tumor-only TMB calculation methods.

## Methods

### Samples and Processing

Molecular data were collected from a cohort of 50 patients with cancer whose tumors were profiled with a 595-gene panel test (xT panel from Tempus Labs Inc) and whose data were available for this analysis.^4^ The xT assay includes a matched tumor and germline sample for analyses. Sample processing, sequencing, alignment, variant calling, and classification were performed as previously described.^5^ Variants called from tumor germline subtraction were considered true somatic variants and were treated as the criterion standard or control group. Variants were evaluated if they had a variant allele fraction of at least 5% and coverage of at least 100× within the tumor. One patient was exchanged from the prior data set for this analysis to ensure all patients had normal samples greater than internal fingerprinting and depth-of-coverage quality standards. Per institutional policy and interpretation of the Common Rule, the use of deidentified data in this project did not warrant institutional review board approval. For this reason, patient consent was not obtained.

### Tumor Mutation Burden Calculation and Variant Grouping

Tumor mutation burden was calculated by adding all missense, insertions/deletions, and frameshift variants within the tumor sample and dividing by the total size of the panel (2.4 Mb). The germline-subtracted TMB measurement was compared with TMB measurements based on 3 variant filtering levels. Level 1 variant filtering (tolerant approach) removed all tumor-only variants with a population allele frequency of at least 1% in the Exome Aggregation Consortium (ExAC) database with the Cancer Genome Atlas (TCGA) cohort removed. This method was selected because of its prior use by other investigators.^6^ Level 2 filtering (stringent approach) removed all variants observed in the non-TCGA ExAC database, simulating a naive approach of removing germline variation. This method was selected to be even more conservative than our first filtering approach. Level 3 filtering (algorithmic approach) used a tumor-only pipeline that classified germline and somatic variants based on tumor purity, sequencing depth, and copy state. The control for the algorithm included a set of 50 unmatched normal samples that allowed the calculation of position quality scores and determination of a mean exon read depth for copy number analysis.^4,7^

### Statistical Analysis

Median TMBs for the germline-subtracted method and each filtering level were calculated. The mean paired differences between each patient’s germline-subtracted TMB and each level of filtering were calculated the epiR package in R, version 3.5.3 (the R Foundation) with the script epi.conf(data, ctype = “mean.paired”, conf.level = 0.95), where data represent a dataframe of the germline-subtracted results and each level of filtering that was compared. We used the DABEST package in R to create the Cumming plot.^8^ A concordance correlation coefficient (*r*) was calculated between the germline-subtracted TMB and the tumor-only TMB of each filtering level using the epiR package in R with the script epi.ccc(x, y, ci = “z-transform”, conf.level = 0.95, rep.measure = TRUE, subjectid), where *x* represents the germline-filtered results and *y* represents each level of filtering.^9^ These analyses were exploratory, and 2-tailed *P *values of less than .05 were considered significant.

## Results

The 50 samples analyzed in this study were from 10 different tumor types (Table). All levels of filtering overestimated TMB compared with germline-subtracted TMB. The median germline-subtracted TMB was 1.7 mutations/Mb (range, 0.4-9.2), while the median TMB for filtering at levels 1, 2, and 3 were 28.8 mutations/Mb (range, 17.5-67.1; paired *P* <.001), 20.8 mutations/Mb (range, 10.4-30.8; paired *P* <.001), and 3.8 mutations/Mb (range, 0.8-12.1 mut/Mb; paired *P* <.001), respectively (Figure). The concordance correlation was weakest for level 1 filtering, which excluded tumor-only variants in the non-TCGA ExAC database with an allelic fraction of at least 1% (*r* = 0.008; 95% CI, −0.004 to 0.020). Removing all non-TCGA ExAC database variants regardless of their allele frequency with our level 2 filtering resulted in better but poor concordance correlation with the control group (*r* = 0.018; 95% CI, 0.003-0.033), while using an algorithmic approach for level 3 filtering improved the concordance correlation further (*r* = 0.54; 95% CI, 0.36-0.68). After overlapping the variants from the different filtering levels with the germline-subtracted variants (data not shown), we found that levels 1 and 3 retained all of the germline-subtracted variants, while level 2 filtering resulted in fewer variants, including the removal of 20% of the germline-subtracted variants.

**Table.** Included Tumor Types

Tumor Type	No. (%)
Brain	4 (8)
Breast	4 (8)
Colorectal	6 (12)
Endometrial	3 (6)
Lung	3 (6)
Ovarian	6 (12)
Pancreatic	4 (8)
Prostate	5 (10)
Other rare tumors	6 (12)
Unknown	9 (18)

**Figure.**
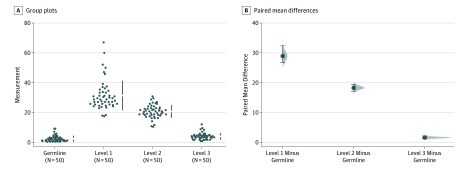
Cumming Plot Showing the Paired Mean Differences in Tumor Mutational Burden Between the Germline-Subtracted Control Group and Filtering Levels 1, 2, and 3 This plot demonstrates the paired mean differences in tumor mutational burden between the germline-subtracted control group and filtering levels 1, 2, and 3. All groups are plotted on the left panel, and each observation is represented by a dot. The paired mean differences are plotted on the right panel as a bootstrap sampling distribution. Each mean difference is depicted as a black dot. The 95% confidence intervals are indicated by the ends of the vertical error bars.

## Discussion

Diverse mutational signatures have been described for several solid tumors, especially for those with underlying carcinogenic or viral exposures.^10^ These mutations potentially give rise to neoantigens that can be detected by the adaptive immune system.^3^ Here, we show that TMB calculation remains to be standardized, and approaches lacking the subtraction of patients’ germline mutations can overestimate the true TMB. While our level 3 classification algorithm to determine TMB resulted in the closest concordance correlation to germline subtraction, it still overestimated TMB in most cases. Historically, whole-exome sequencing was used to calculate TMB, and targeted sequencing panels were later validated to correlate with whole-exome sequencing for TMB calculation.^11^ However, most commercial platforms use custom gene panels that have not been standardized. Subsequently, algorithm-based TMB calculations not requiring normal tissue for germline subtraction were developed.^12,13^ While some platforms now include germline mutation subtraction using patients’ normal tissue or blood, others do not.

A 2019 report^14^ demonstrated that TMB predicts survival after immunotherapy for several cancer types, but the definition of high TMB varied between malignant neoplasms.^14^ For example, the cutoff level for high TMB in colorectal cancer was 52.2 mutations/Mb, while the cutoff level in lung cancer was 13.8 mutations/Mb. This variability across tumor types makes the standardization of testing across different platforms even more critical. We postulate that individualized germline data will help further the development of TMB as a biomarker. Another 2019 study^15^ showed that chromosomal rearrangements resulting in chromothripsis and chromoplexy have neoantigenic potential.^15^ These complex events can be detected using mate-pair sequencing and RNA sequencing but might be missed using targeted gene panels,^16^ thus resulting in a falsely low TMB. Furthermore, the presence of a DNA mutation does not guarantee that a mutant protein will be translated, processed, and presented on a major histocompatibility complex protein. Overall, these findings suggest that accurate TMB determination may require more than 1 sequencing method.

### Limitations

Our study was limited by the sample size. It is possible that filtering approaches may have stronger associations than we observed in specific tumor types. With more cases, different filtering approaches, or population databases there may be better associations. Also, we did not make adjustments for tumor purity, which may also be associated with TMB.

## Conclusions

Although our study population of 50 patients is relatively small and lacks data on treatment outcomes, it is the first study, to our knowledge, to identify significant discordance in TMB calculations between germline variant subtraction, population filtering, and algorithmic approaches. Our results demonstrate that TMB determined from tumor-only sequencing can differ significantly from TMB determined by paired tumor-normal sequencing. Despite the improved associations observed with more stringent filtering approaches, estimates based on tumor-only sequencing may result in the inaccurate categorization of tumors, with downstream negative effects on clinical trials and patient outcomes.
